# Diversity of trypanosomes in wildlife of the Kafue ecosystem, Zambia

**DOI:** 10.1016/j.ijppaw.2020.04.005

**Published:** 2020-04-23

**Authors:** David Squarre, Kyoko Hayashida, Alex Gaithuma, Herman Chambaro, Naoko Kawai, Ladslav Moonga, Boniface Namangala, Chihiro Sugimoto, Junya Yamagishi

**Affiliations:** a Research Center for Zoonosis Control, Hokkaido University, Kita-Ku, Sapporo, Hokkaido, 001-0020, Japan; b Wildlife Veterinary Unit, Department of National Parks and Wildlife, P/Bag 1, Chilanga, Zambia; c The Royal (Dick) School of Veterinary Studies, University of Edinburgh, Easter Bush Campus, EH25 9RG, UK, United Kingdom; d Central Veterinary Research Institute, P.O Box, 33980, Chilanga, Zambia; e Department of Paraclinical Studies, University of Zambia, P.O. Box 32379, Lusaka, 10101, Zambia; f Global Station for Zoonosis Control, GI-CoRE, Hokkaido University, Kita-ku, Sapporo, Hokkaido, 001-0020, Japan

**Keywords:** *Trypanosoma brucei rhodesiense*, Reservoir, Kafue national park

## Abstract

The Kafue ecosystem is a vast conservation protected area comprising the Kafue National Park (KNP) and the Game Management Areas (GMA) that act as a buffer around the national park. The KNP has been neglected as a potential foci for rhodesiense sleeping sickness despite the widespread presence of the tsetse vector and abundant wildlife reservoirs. The aim of this study was to generate information on circulating trypanosomes and their eminent threat/risk to public health and livestock production of a steadily growing human and livestock population surrounding the park. We detected various trypanosomes circulating in different mammalian wildlife species in KNP in Zambia by applying a high throughput ITS1-polymerase chain reaction (PCR)/nanopore sequencing method in combination with serum resistant associated-PCR/Sanger sequencing method. The prevalence rates of trypanosomes in hartebeest, sable antelope, buffalo, warthog, impala and lechwe were 6.4%, 37.2%, 13.2%, 11.8%, 2.8% and 11.1%, respectively. A total of six trypanosomes species or subspecies were detected in the wildlife examined, including *Trypanosoma brucei brucei*, *T. godfreyi*, *T. congolense*, *T. simiae* and *T. theileri*. Importantly we detected human infective *T. b. rhodesiense* in buffalo and sable antelope with a prevalence of 9.4% and 12.5%, respectively. In addition, *T. b. rhodesiense* was found in the only vervet monkey analyzed. The study thus reaffirmed that the Kafue ecosystem is a genuine neglected and re-emerging foci for human African trypanosomiasis. This is the first assessment of the trypanosome diversity circulating in free-ranging wildlife of the KNP.

## Highlights

• Detected six African trypanosomes in wildlife species of Kafue National Park using ITS1-PCR and Nanopore sequencing method.
• Confirmed presence of *Trypanosoma brucei* rhodesiense using SRA PCR.
• Identified unique divergence of SRA sequence of *Trypanosoma brucei rhodesiense* from buffalo, sable and vervet monkey.

## Graphical abstract

A herd of wild African buffalo maintaining the circulation of trypanosomes in tsetse fly populated Kafue ecosystem.

**Figure:**
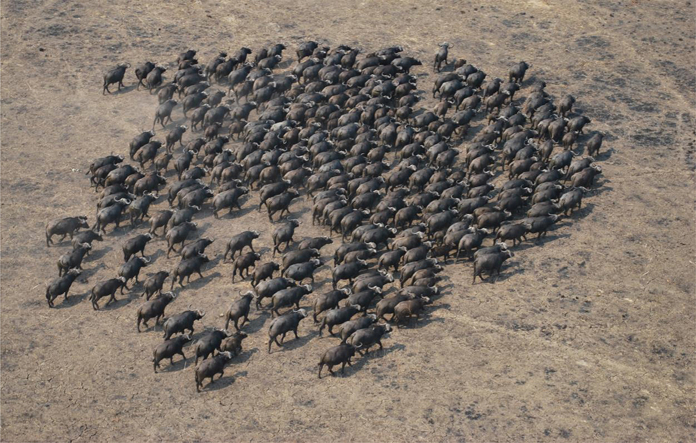

## 1 Introduction

African trypanosomiasis affects both human and livestock populations in sub-Saharan Africa, resulting in measurable socio-economic and public health impacts, especially in poor rural communities (Mwiinde et al., 2017; Simwango et al., 2017; World Health Organization [WHO], 2015a). The disease is caused by a multi-host hemoflagellate protozoan parasite of the genus *Trypanosoma* and is transmitted by infected tsetse flies (*Glossina* spp.) (World Health Organization, 2019). The subspecies of *Trypanosoma brucei rhodesiense* and *Trypanosoma brucei gambiense* are responsible for human African trypanosomiasis (HAT), which is also known as sleeping sickness (Deborggraeve et al., 2008). In Zambia, HAT resulting from *T. b. rhodesiense* (rHAT) accounts for all sleeping sickness cases (Anderson et al., 2015; World Health Organization, 2017). The species of trypanosome that cause animal African trypanosomiasis or nagana in livestock includes *T. brucei*, *T. congolense, T. simiae,* and *T. vivax* (Simukoko et al., 2011). Other species, such as *T. godfreyi* have unknown pathogenicity, while *T. theileri* is non-pathogenic. The *T. theileri* can also be spread by other species of biting flies besides tsetse fly. The inherent foci and circulation of rHAT and nagana in Zambia follow an endemic vector distribution mostly in conservation areas and surrounding areas (Van den Bossche et al., 2010). Wildlife in these conservation areas serve as animal reservoirs for rHAT. Current conservation strategies aimed at increasing wildlife populations in conservation areas (Department of National Parks and Wildlife, 2018; Government of Zambia, 2015; Ministry of National Development Planning, 2017) favor the enrichment of circulating parasites through the elaborate wildlife/tsetse fly interactions.

Conservation areas preserve and protect the environment and important ecological/biodiversity hotspots that maintain ecosystem services (Fanin et al., 2018). Interactions of vectors that transmit the parasite, an abundance of diverse wildlife reservoirs and an accommodating ecology play important roles in sylvatic transmission dynamics and the sustained circulation of the parasite (Auty et al., 2016). Encroachment of human developments and migration of people and their livestock into conservation areas can create, extend or intensify the scale of the existing interface within conservation areas (Bengis et al., 2002; Mweempwa et al., 2015; Stoddard et al., 2009). This has led to increasingly frequent encounters between the vector and human communities, facilitating the spillover of infection from wildlife reservoirs into the human populations and livestock. More than any other disease, trypanosomiasis is closely associated with the conservation of biodiversity (Anderson et al., 2015).

Blood meal analysis has been used to identify tsetse fly host preferences and ascertain reservoir communities. The two major species found in Kafue National Park (KNP) are *Glossina morsitans centralis* and *G. pallidipes*. Although host preferences are highly dependent on host availability, suids and bovids are considered probable favorite host for *G. morsitans* and *G. pallidipes* (Clausen et al., 1998; Leak, 1999). Suids, bovids, and primates have been also reported to be blood meal sources for *G. m. morsitans* in Zambia (Okiwelu, 1977).

Molecular identification of trypanosome species and subspecies is often based on polymerase chain reaction (PCR) amplification of ribosomal RNA sequences of the internal transcribed spacer 1 (ITS1) of the small ribosomal subunit of 18S and 5.8S (Njiru et al., 2004). Recently developed primers and next generation sequencing (NGS) using unique barcodes have been shown to be more sensitive methods of identifying trypanosomes (Gaithuma et al., 2019). As the subgenus *Trypanozoon* has the same length of the ITS1 product, PCR targeting the human serum-resistant associated (SRA) gene has been used to identify human infective *T. b. rhodesiense* (Gibson et al., 2002).

Although efforts to eliminate of gambiense HAT (gHAT) are making progresses, rHAT elimination is proving difficult due to the presence of wildlife and domestic reservoirs. The host range and distribution of reservoir populations should be considered in further studies. Cases of rHAT in Zambia, have traditionally been recorded in Luangwa Valley and the Lower Zambezi ecosystem (Munang'andu et al., 2012). Recently, the KNP recorded the first human case of rHAT after almost half a century (Squarre et al., 2016). Historically, the KNP has been neglected as a potential focus for rHAT despite the widespread presence of the vectors *G. morsitans centralis* and to a less extent *G. palpalis* (Food and Agriculture Organization of the United Nations, 1992). The area has been considered devoid of the parasite due to a lack of compelling data on the presence, abundance, and diversity of the circulating parasites, particularly in wildlife reservoir populations.

The Kafue ecosystem is a vast conservation area covering approximately 68,000 km^2^. It comprises two types of protected areas; the national park itself and game management areas (GMA) that serve as a buffers around the park. The KNP is a reserve set aside for nature and biodiversity conservation. Only activities such as photographic tourism that pose a minimal risk of disturbance or threat to the landscape, fauna, and flora are sanctioned. Undertakings or land use activities that do not conform to or promote the intrinsic value of the park, such as human settlement, hunting, agriculture/livestock, mining, or logging, are not permitted in the confines of the KNP (Zambia Wildlife Authority, 2011a). However, the nine GMAs surrounding the park allow the proximate cohabitation of wildlife and people. Anthropogenic activities, such as human settlement, hunting, agriculture, infrastructure development, and fishing, are permissible and have been streamlined in land use plans that integrate and optimize wildlife conservation and sustainable socio-economic utilization of natural resources by the communities that live in the GMAs (Zambia Wildlife Authority, 2013a, 2013b). The presence and co-existence of wildlife, tsetse flies, humans, and their livestock makes GMAs typical human-wildlife-livestock-tsetse fly interface areas in distinct contrast to the national park, which is characterized by an elaborate wildlife-tsetse fly interaction zone. Problems involving trypanosomiasis associated with the interface in the Kafue ecosystem were realized decades ago, as demonstrated by the closure of the Itumbi safari camp in 1956 due to sleeping sickness. In 1972, the tsetse control services cleared vegetation, eliminated wildlife, conducted aerial insecticide spraying, and constructed a game fence in the Nkhala area on the southeastern border of the KNP to address trypanosomiasis problems arising from the interface and interrupted the interaction of wildlife with tsetse flies and communities with their livestock (Clarke, 1974; Mwima, 2001; Steel and Glendhil, 1982).

This study characterizes the nature of trypanosomes circulating in the wildlife reservoir community in the KNP and the potential risk of spillovers to human and livestock populations via wildlife. The study employed a MinION-based high-throughput ITS1-PCR/NGS system (Gaithuma et al., 2019). MinION is a transportable and affordable sequencing device designed for field use, that is applicable to epidemiological studies.

## 2 Methods

### 2.1 Study location and sample collection

Sample were collected in 2017 and 2018 in the KNP. The Kafue ecosystem is a large conservation area located in central Zambia (between 14°03″S and 16°43″S and 25°13″E and 26°46″E) comprising the 22,400 km^2^ of parkland (Zambia Wildlife Authority, 2011b) and 45,406 km^2^ of GMAs surrounding the park (Zambia Wildlife Authority, 2013c, 2013d, 2013e, 2013f, 2013a, 2013b, 2013g, 2013h). Blood samples were opportunistically collected from wild animals immobilized or captured for the purpose of (i) clinical interventions, (ii) placement of very high frequency (VHF)/global positioning system (GPS) collars to track spatial movements, and (iii) translocations to other wildlife estates within Zambia. Immobilization of the animals followed protocols and methods as described by Kock and Burroughs (2012) and La Grange (La Grange, 2006).

**Fig. 1:**
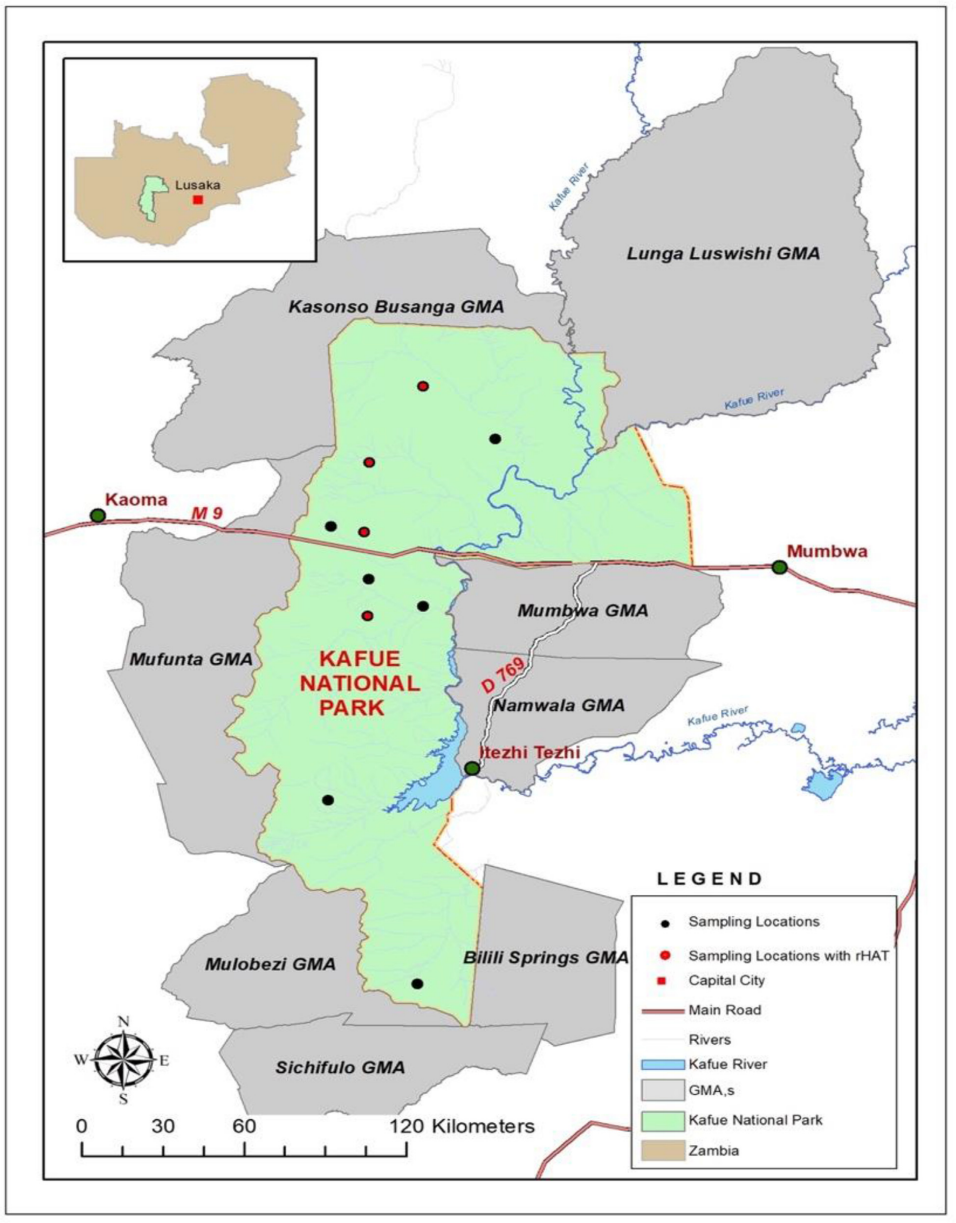
The Kafue ecosystem comprising of the KNP and surrounding GMAs. The black spots indicate sampling points and the red spots indicate areas where rHAT was detected. (For interpretation of the references to colour in this figure legend, the reader is referred to the Web version of this article.)

Venous blood samples were aseptically collected by venipuncture using 5 ml syringes and sterile 18G or 21G needles via the jugular or ear veins following chemical immobilization and physical restraint. Blood samples were collected in ethylenediaminetetraacetic acid (EDTA) tubes from 248 free-ranging wild animals comprising ten mammalian wildlife species. Immediately after the collection, the samples were placed in a portable refrigerator at a temperature of 4 °C and later transported to the laboratory, where they were stored at −80 °C until analysis. All wild animals immobilized in 2017 and 2018 were included in this study. The GPS positions were recorded for all sampling locations. All samples were collected between the months of May and September of each year. ArcView implemented in ArcGIS was used to make spatial illustrations of the sampling point distribution on the map presented in Fig. 1.

### 2.2 Ethical clearance

The blood samples used in this study were collected from free-ranging wild animals in the KNP with the authority from and permits issued by the Department of National Parks and Wildlife, Zambia (TJ/NPW/8/27/1). Ethical clearance for this work was obtained from the Excellence in Research Ethics and Science (ERES) Converge in Zambia (Ref. No. 2019-Jul-010).

### 2.3 DNA extraction

Genomic DNA was extracted from the whole blood samples using a DNA isolation kit for mammalian blood (Roche Applied Science, Indianapolis, USA). A 200 μL sample of DNA was eluted in Eppendorf tubes and stored at −80 °C until further analysis.

### 2.4 ITS1-PCR and species confirmation by MinION sequencing

**Fig. 2:**
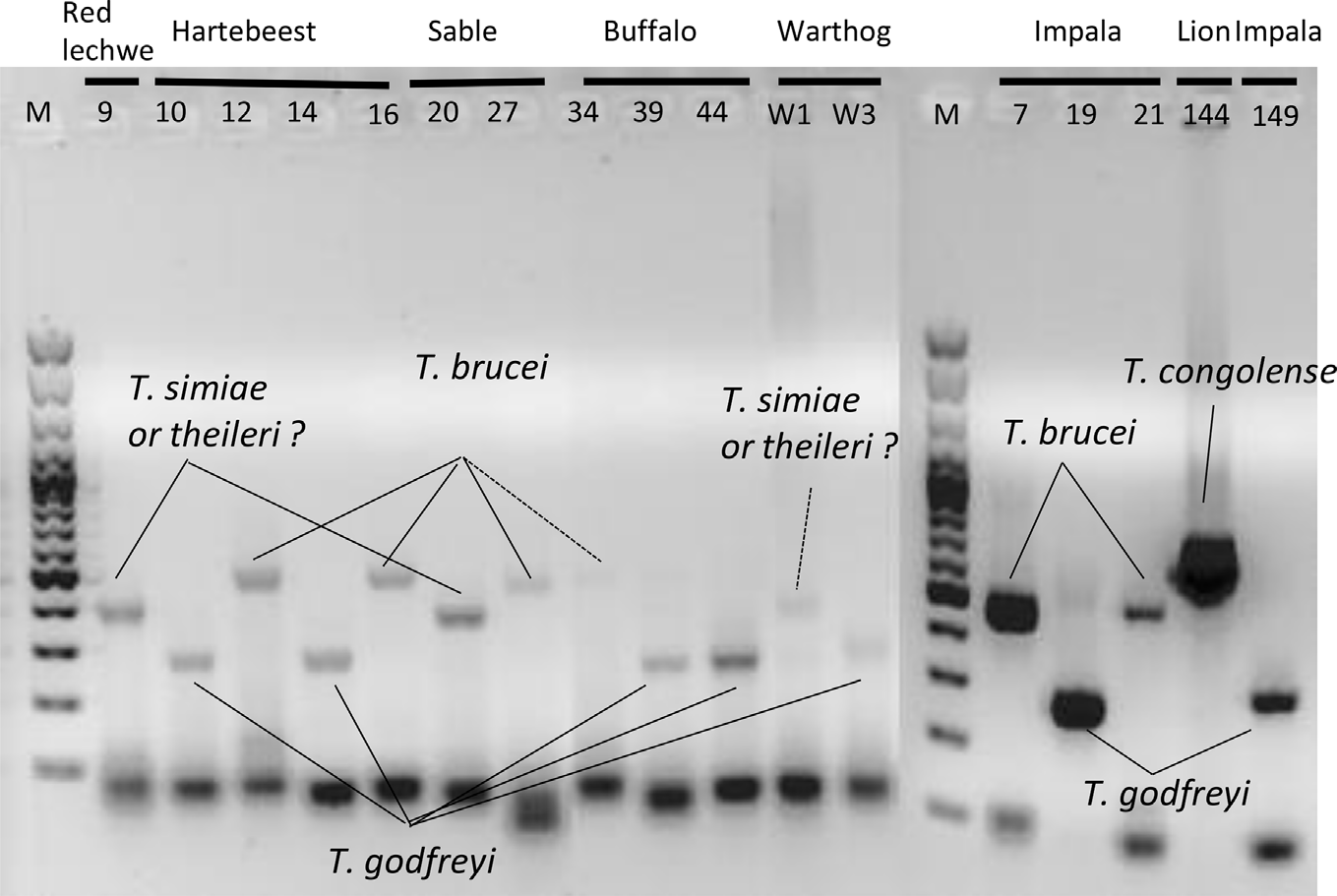
ITS1-PCR Gel analysis Gel image of the ITS-PCR-positive samples. The species were estimated by the band size. The expected sizes for each species are *T. godfreyi*: 220 bp, *T. simiae*: 331–343 bp, *T. theileri*: 269–350 bp, *T. brucei*: 415–431 bp, *T. congolense*: 560–705 bp (Gaithuma et al., 2019).

A modified ITS1-PCR described by Gaithuma (Gaithuma et al., 2019), was used to identify and distinguish clinically infective trypanosome species and subspecies (Table S1). The PCR reaction was mixed in a 10 μL scale comprised 5.0 μL of Ampdirect plus buffer (Shimadzu, Kyoto, Japan), 0.05 μL of BioTaq HS (Bioline, London, UK), 0.2 μL of 2% dimethyl sulfoxide (DMSO), 2.25 μL of nuclease free water, 2 μL of eluted DNA as a template, and 0.25 μL each of 10 μM AITS primers as described by Gaithuma. Amplification conditions involved an initial denaturation step at 95 °C for 10 min followed by 40 cycles of denaturation at 94 °C for 30 s, annealing at 57 °C for 1 min, an extension step at 72 °C for 2 min, and a final extension at 72 °C for 10 min. The PCR products were loaded onto 1.5% agarose gel containing GelRed nucleic acid stain (Biotium, Fremont, CA, USA) and the separated products were visualized under ultraviolet (UV) light in a transilluminator as presented in Fig. 2.

The 22 samples that exhibited a positive reaction by ITS1-PCR and four additional samples with ambiguous signals were validated by MinION sequencing. For multiplex sequences, in-house unique index sequences were added at both terminals by PCR. The index-PCR reaction was conducted by adding 1/60 dilution of the first PCR product into a 10 μL reaction mix with the index primers listed in Table S1. The reaction and thermocycler conditions were similar to the above, except that amplification was for 15 cycles.

PCR amplicons after indexing were pooled to 12, and each pool was subjected to library construction using a ligation sequencing kit and native barcoding kit 1D (SQK-LSK109 and EXP-NBD103, respectively; Oxford Nanopore Technologies, Oxford, UK) according to the manufacturer's instruction. Sequencing was conducted using FLO-MIN106 (Oxford Nanopore Technologies). The obtained fast5 was converted to fastq and de-barcoded by Albacore (Oxford Nanopore Technologies). After de-indexing was performed by custom scripts based on the alignment score from the obtained sequences and indexed primers using LAST (Kielbasa et al., 2011), the obtained de-multiplexed sequences were then examined by BLAST against a nucleotide database to confirm the infected species. The best hit sequence from BLASTn analysis was retrieved. A species was discriminated if more than 20% of the total reads amounting to at least 50 reads was identified. Judgments of the MinION sequencing analysis are presented in Table 1.

**Table 1:** The MinION sequence results of ITS-PCR amplicons.

ID #	Wildlife Species	Total reads obtained^a^	Hit reads^b^ (%)	Trypanosome species identification by MinION
**9**	Red lechwe	556	289 (52.0%)	*T. theileri*
**10**	Hartebeest	8830	7062 (80.0%)	*T. godfreyi*
**12**	Hartebeest	467	278 (59.5%)	*T. brucei*
**14**	Hartebeest	3138	2515 (80.1%)	*T. godfreyi*
**16**	Sable antelope	65	54 (83.1%)	*T. brucei*
**20**	Sable antelope	6319	3762 (59.5%)	*T. theileri*
**27**	Sable antelope	93	55 (59.1%)	*T. brucei*
**34**	Buffalo	302	93 (30.8%)	*T. brucei*
**39**	Buffalo	547	439 (76.5%)	*T. godfreyi*
**44**	Buffalo	2336	1956 (83.7%)	*T. godfreyi*
**W1**	Warthog	447	136/181 (30.4%/40.5%)	*T. godfreyi/T. Simiae*
**W3**	Warthog	571	412 (72.2%)	*T. godfreyi*
**7**	Impala	482	454 (94.2%)	*T. brucei*
**19**	Impala	24,213	11272 (46.6%)	*T. godfreyi*
**21**	Impala	139	110 (79.1%)	*T. brucei*
**144**	Lion	8221	7886 (46.6%)	*T. congolense*
**149**	Wild dog	1047	822 (78.5%)	*T. godfreyi*

a The total read number obtained after de-indexing.

b The obtained reads were blasted against BLASTn database, and the read number of the top hit are shown.

### 2.5 SRA-PCR and sequencing analysis

To detect human infective *T. b. rhodesiense*, PCR amplifying partial 284 base pairs (bp) of the SRA gene (Radwanska et al., 2002) was conducted by adding 2 μL of a DNA template to a 10 μL reaction mix comprising of 0.05 μL of BioTaq HS, 5 μL of Ampdirect Plus buffer, 2.55 μL nuclease free water and 0.2 μL each of the SRA primers in Table S1. The thermocycler conditions consisted of an initial denaturation at 95 °C for 10 min and 40 cycles of denaturation at 94 °C for 30 s, annealing at 60 °C for 1 min, and extension at 72 °C for 2 min, and final extension at 72 °C for 5 min. The PCR products were loaded onto a 2% agarose gel stained with GelRed nucleic acid stain and visualized in a UV transilluminator. The PCR products were purified by ExoSAP-IT (GE Healthcare/USB, USA) following the manufacturer's instructions. Purified PCR products were sequenced using BigDye Terminator version 3.1 (Applied Biosystems, Foster city, CA, USA) on an automated capillary sequencer (Applied Biosystems 3130 Genetic Analyzer; Applied Biosystems Japan Ltd., Tokyo, Japan). Obtained sequences were analyzed by Molecular Evolutionary Genomics Analysis version 7 (MEGA 7) (Kumar et al., 2016) and aligned with the three reference SRA sequences from Uganda (AF097331), Zambia (AJ345058) and Kenya (AJ345057) using MEGA 7.

## 3 Results

### 3.1 Prevalence and polymorphism of trypanosome infection in wild animals

ITS1-PCR revealed that 17 wildlife animals were found to be infected by *Trypanosoma* spp. The infective *Trypanosoma* spp. were inferred by gel electrophoresis, based on the product size range (Fig. 2). In the ITS1-PCR used in this study, differentiation between *T. simiae* and *T. theileri* by the gel was impossible due to the overlapping product sizes (*T. simiae*: 331–343 bp and *T. theileri*: 269–350 bp). For definitive species identification of the ambiguous samples, a MinION sequencing was conducted. The results agreed with the inferred trypanosome species by gel analysis and further definitively differentiated *T. simiae* and *T. theileri*. Three samples with bands in the *T. simiae/T. theileri* range (Fig. 2), were differentiated into two species *T. theileri* in lechwe (*Kobus leche leche*) #9, and sable antelope (*Hippotragus niger*) #20, and *T. simiae* in warthog (*Phacochoerus africanus*) #W1 by sequence analysis, providing more discriminative power than gel analysis.

Based on the SRA-PCR analysis, three species of vervet monkey (*Chlorocebus pygerythrus*), sable antelope and buffalo (*Syncerus caffer*) were determined to have been infected with the human infective *T. b. rhodesiense* (Table 2)*.* Among them, sable antelopes and buffalos exhibited the highest prevalence of the infection (12.5% and 9.4%, respectively). The species that was most infected by a wider diversity of trypanosome species or subspecies was also the buffalo, which was infected by three different trypanosome species or subspecies (*T. b. rhodesiense*, *T. b. brucei and T. godfreyi*).

**Table 2:** Summary of trypanosome subspecies/species diversity in mammalian wildlife species in Kafue National Park.

Species	Population estimate in KNP^b^	Number (n)	ITS1-PCR/NGS				ITS1-PCR/NGS and SRA-PCR		Trypanosome prevalence in wildlife species (%)
			T. congolense	***T. simiae***	T. godfreyi	T. theileri	T. b brucei	T. b. rhodesiense	
Hartebeest	6265	47			2		1		6.4
Sable antelope	14,314	8				1	1	1(1)^a^	37.5
Buffalo	8534	53			1		1	5(0)^a^	13.2
Vevert Monkey	N/A	1						1(0)^a^	100
Warthog	9143	17		1	1				11.8
Lion	N/A	4	1						25
Wild dog	N/A	2			1				50
Impala	25,847	106			1		2		2.8
Cheetah	N/A	1							0
Red Lechwe	12,290	9				1			11.1

a Numbers between brackets represent the number of heads positive for both ITS-PCR/NGS and SRA-PCR.

b Department of National Parks and wildlife, 2016

Ideally, SRA positive samples should be a subset of *Trypanozoon* identified by ITS1-PCR. Accordingly, one sable antelope was both ITS1-PCR and SRA-PCR positive. However, some discrepancies remain between ITS1-PCR and SRA-PCR in this study. For instance, according to our data, five buffalos and one vervet monkey were *T. b. rhodesiense*-positive (SRA-PCR-positive) but results of ITS1-PCR test were negative. This discrepancy may be due to the higher sensitivity of SRA-PCR than ITS1-PCR. To test this assumption, we conducted a detection-limit determination assay using *T. b. rhodesiense* IL1501 pure DNA and verified that SRA-PCR is more sensitive than ITS1-PCR (Supplemental Data 3). We concluded that a species was *T. b. rhodesiense* if SRA-PCR was positive regardless of the ITS1-PCR results and *T. b. brucei* if SRA-PCR was negative but ITS1-PCR-positive in each sample.

### 3.2 Diversity of SRA gene sequences

**Fig. 3:**
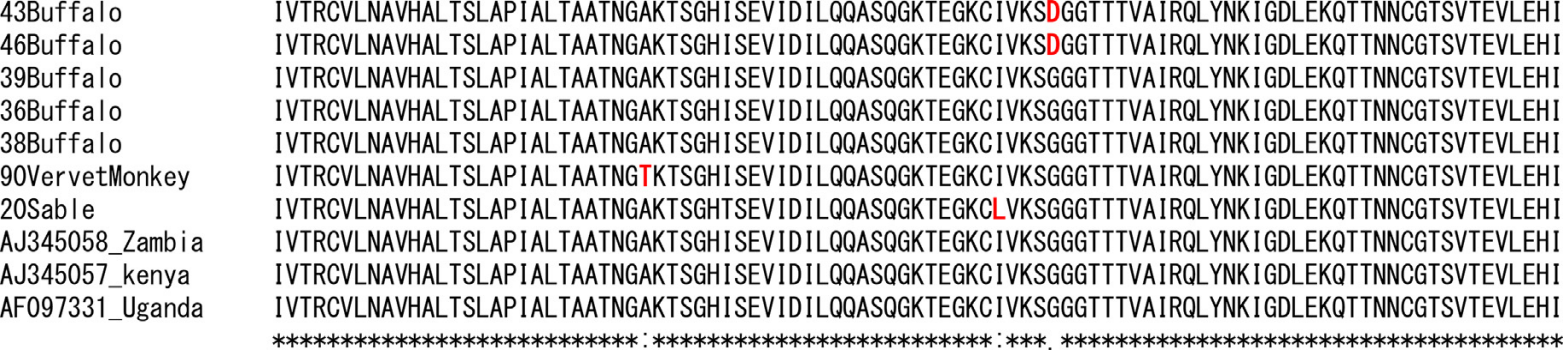
The sequences of SRA-PCR–positive samples from buffalo, sable antelope, and vervet monkey are determined and the deduced amino acid were aligned with deposited representative sequences from Zambia, Kenya, and Uganda.

The results reveal a discernible substitution of the thymine for adenosine on the SRA sequence obtained from the vervet monkey. Another substitution of adenosine for guanine was observed on two SRA sequences from two buffalos. All substitutions on the SRA nucleotide sequences altered the codons, resulting in variation in the translated amino acid sequences. The amino acid alanine was substituted by threonine, glycine by aspartic acid and isoleucine by leucine in the sequence from the vervet monkey, buffalos and sable antelopes, respectively, resulting in divergence from the reference sequences from Uganda, Zambia, and Kenya (Fig. 3). The obtained divergent partial SRA nucleotide sequences obtained are available at GenBank under the accession number MN635739 (vervet monkey), MN635743 and MN635744 (buffalo) and MN635738 (sable antelope).

## 4 Discussion

This work is the first assessment of trypanosomes circulating in free-ranging wildlife in the KNP based on blood samples collected from mammalian species. The study established a diverse array of trypanosome species in the park's wildlife populations. The samples were all collected in the KNP precincts and in habitats distant from human settlement or livestock. Although there is a marked distinction between the national park and the GMAs in terms of conservation management and goals, collectively they form a large and uninterrupted conservation area with open borders that do not in any way hinder the movement of either vectors or wildlife reservoirs between the park and GMAs. The free movement of wildlife host and the vectors implies a continuous risk of possible spillover of trypanosomes from the park to the growing human and livestock populations in the GMAs. More than 200,000 people and their livestock permanently settled in the GMAs are exposed to this risk, which compounds and enriches the human-wildlife-livestock-tsetse fly interface in the GMA. Both wildlife and increasing livestock numbers in the GMAs form a reservoir community. The role of livestock as a potential reservoir remains to be investigated.

The largest threat to conservation areas is the continuous increase in human migration and encroachment (Waldron et al., 2017; Wittemyer et al., 2008), especially into GMAs and up to the border of the park, creating ecological mosaics that could lead to increased and frequent exposure and interactions between the parasite and human/livestock populations occurring at different scales of the human-wildlife-livestock-tsetse fly interface. Those likely to be exposed to infection include the park's annual 11,250 photographic tourist visitors, the more than 200,000 people who live in all the GMAs surrounding the park (Zambia Wildlife Authority, 2013c, 2013d; 2013e, 2013f; 2013a, 2013b; 2013g, 2013h), park officials and staff, and a large numbers of people who use the main roads (M9 and D769) that intersect the park and GMAs.

Convenience sampling was employed in this study due to the numerous challenges involved in collecting biological samples from free-ranging wild animals (Bengis et al., 2002). Because most of the samples were from animals immobilized for restocking or breeding programs, sex ratios favored females, and age distribution favored adults of breeding ages. However, sex and age do not affect the prevalence of trypanosomes in wildlife (Anderson, 2008). There are 158 wildlife species of large and small mammals in the KNP (Zambia Wildlife Authority, 2011a). Ten major species are represented in this study and the total number of estimated heads is 76,494 (Department of National Parks and wildlife, 2016) excluding lions, wild dogs and vervet monkeys, estimates for which are not available. Other important species that were not included in the study are kudu (*Tragelaphus strepsiceros*), waterbuck (*Kobus ellipsiprymnus*), eland (*Taurotragus oryx*), and puku (*Kobus vardonii*). Their estimated numbers are 1251, 7261, 1156, and 16470, respectively (Department of National Parks and wildlife, 2016). As they are relatively minor populations compared with the tested species; our study represents the major part of the ecosystem. Nevertheless, further less-biased studies should be conducted.

A total of six trypanosome species or subspecies (T*. b. rhodesiense, T. godfreyi, T. b. brucei, T. congolense, T. simiae,* and *T. theileri*) were detected using a combination of molecular techniques of ITS1-PCR/NGS and SRA-PCR/Sanger sequence analyses. ITS1-PCR/NGS is more sensitive and offers greater accuracy when diagnosing a wide range of trypanosomes comparing with conventional ITS1-PCR gel analysis, which produces relatively imprecise identification of trypanosomes based on band size (Gaithuma et al., 2019). However, the former method cannot discriminate among Trypanozoon subspecies due to the species's highly conserved genome (Cuypers et al., 2017), and thus SRA-PCR and Sanger sequence analysis were conducted in addition to ITS1-PCR/NGS to identify the important human infective trypanosome *T. b. rhodesiense*. The discrepancies observed in this study between ITS1-PCR and SRA-PCR can be attributed to the high sensitivity of SRA-PCR relative to ITS1-PCR, as demonstrated in the detection-limit determination assay (Supplementary Data 3). The low and persistent phases of parasitemia frequently seen in wildlife (Laohasinnarong et al., 2015; Van Den Bossche et al., 2005) can be problematic in detecting the parasites.

Trypanosomes that cause disease in livestock were also detected, including *T. congolense, T. b. brucei and T. simiae.* The wide-ranging livestock in the GMAs would usually and instantaneously share and access the same pools of resources, such as water and pasture, facilitating the exchange of diseases including trypanosomiasis. Generally, nagana is a major hindrance to livestock production in tsetse-inhabited areas and wildlife reservoirs most likely compounds this problem.

In the last 50 years, no specific rHAT surveillance in KNP has been undertaken to diagnose the disease or demonstrate its presence. Because rHAT is not pathognomonic and its symptoms are similar to those of many other febrile conditions, it is likely that the disease has been misdiagnosed and masked by malaria and other common diseases. The recent focus on HIV/AIDS, malaria, and tuberculosis, and the widespread deviation from the routine use of microscopy due to increased use of rapid detection test kits for diseases such as malaria, may have hampered the proper diagnosis of rHAT (Mwanakasale and Songolo, 2011; Namangala et al., 2012).

This study revealed substantial infection rates of *T. b. rhodesiense* (12.5% in sable antelopes and 9.4% in buffalos) in the wildlife population of the KNP and further supports the recent diagnosis of *T. b. rhodesiense* in an adult male patient from the KNP using loop isothermal mediated amplification, which demonstrated the presence of rHAT in the KNP (Squarre et al., 2016). The outcomes and results of this study confirm the presence of rHAT in the KNP and further confirm that the KNP is a genuine neglected and re-emerging focus of rHAT. The first step to control this disease is to acknowledge its presence and the potential risk it presents. Based on the results reported here, it is recommended that the already existing and accessible health facilities should be bolstered with the capacity to diagnose and treat rHAT within and around the KNP (Holmes, 2015).

Buffalo had the highest infection rates of *T. b. rhodesiense* in this study. The gregarious nature of buffalo in the KNP leads to the formation of large herds of 20–200 animals. Their ability to traverse different habitats combined with the presence of the disease vector could explain the ease of spread and maintenance of trypanosome parasites. The *T. b. rhodesiense* infection rate in buffalo in this study was consistent with similar studies from the Luangwa Valley that reported significantly high infection rates in buffalos (Anderson et al., 2011). This is also consistent with tsetse fly host preferences from blood meal analysis conducted in Zambia (Clausen et al., 1998). In contrast, the presence of *T. b. rhodesiense* in vervet monkey in this study deviated from the results of comparable studies in the Luangwa Valley that reported no trypanosome infections in these non-human primates (Anderson et al., 2011; Nakayima et al., 2014). Vervet monkeys are not well recognized as a common blood source for tsetse flies, though there are a few reports of this species’ DNA having been detected in *G. morsitans* using blood meal analysis (Clausen et al., 1998). Vervet monkeys are also known to be the susceptible hosts for *T. b. rhodesiense* infection in experimental models (Thuita et al., 2008). The significance of vervet monkey as a natural rHAT reservoir should be considered for further assessment.

Particular attention should be paid to the interface that involves buffalo due to the risk of the tsetse fly vector passing on the infection to livestock and humans at points where they share common pool resources such as water, pasture and habitat. Non-human primates such as the vervet monkey tend to cause a specific human-wildlife conflict, mostly due to the monkey's tendency to wander into human dwellings in search of unsecured and discarded food and harvest. Such interactions provide an opportunities for monkeys to serve as sources of infection for the vector. This assessment broadens the basic information to help in predicting disease risk due to the likely spillover of the parasites from wildlife into human/livestock populations and its larger implications.

The aligned sequence of the SRA gene from the vervet monkey, sable antelope, and buffalo showed slight differences in the nucleotide sequences relative to the reference sequences from Zambia (AJ345058), Kenya (AJ345057) and Uganda (AF097331). The divergence of the SRA nucleotide sequence translates into corresponding diversity in the alignment of amino acid sequence as revealed by the SRA primary structure of protein. The immediate implication of this diversity in terms of the functional significance of the trypanolytic effect on human serum was not evaluated, but this diversity may be epidemiologically significant.

This study used NGS by Oxford Nanopore Technologies MinION to validate the ITS1-PCR amplicons, which produced reliable nucleotide sequences in real-time at relatively little cost. This approach can be extended to field diagnosis of wildlife-associated diseases (anthrax, rabies, foot-and-mouth disease, African swine fever) (Hansen et al., 2019) and the molecular identification of wildlife species to meet wildlife forensics and intelligence needs in combating the illegal wildlife trade and trafficking (Johnson et al., 2014).

The WHO considers rHAT a public health risk that should be eliminated by 2030 (Franco et al., 2018). This can be realized through a multi-sectorial One Health approach that integrates contribution by medical and veterinary professionals, social scientist, and wildlife officials. The rHAT risks are influenced by wildlife distribution, habitat management and land use. A more holistic ecological approach should be advanced (World Health Organization (WHO), 2015b).

In summary, the molecular tests employed in this study revealed that trypanosomes parasites are circulating in wildlife reservoirs while human and livestock populations in and around the Kafue ecosystem expand. We also detected a human infective/zoonotic trypanosome, *T. b. rhodesiense*, further suggesting that the KNP is a neglected and re-emerging focus of rHAT.

## Data availability statement

The SRA sequences obtained by Sanger sequence are deposited in GenBank **MN635738-MN635744.**

## Funding

This study was funded by the 10.13039/501100001691Japan Society for the Promotion of Science Ronpaku Program (JSPS) Japan, The 10.13039/501100009036International Collaborative Research Program for Tackling NTD (Neglected Tropical Disease) Challenges in African countries (JP18jm0510001) Japan, and the 10.13039/100009619Agency for Medical Research and Development (AMED), Japan.

## Declaration of competing interest

None.
