# Supplementary material for: Diversity of trypanosomes in wildlife of the Kafue ecosystem, Zambia
Source: Int J Parasitol Parasites Wildl. 2020 Apr 23;12:34–41. doi: 10.1016/j.ijppaw.2020.04.005 (PMC7215119; doi:10.1016/j.ijppaw.2020.04.005)
Supplement: Multimedia component 1 [file mmc1.pptx]

## Slide 1
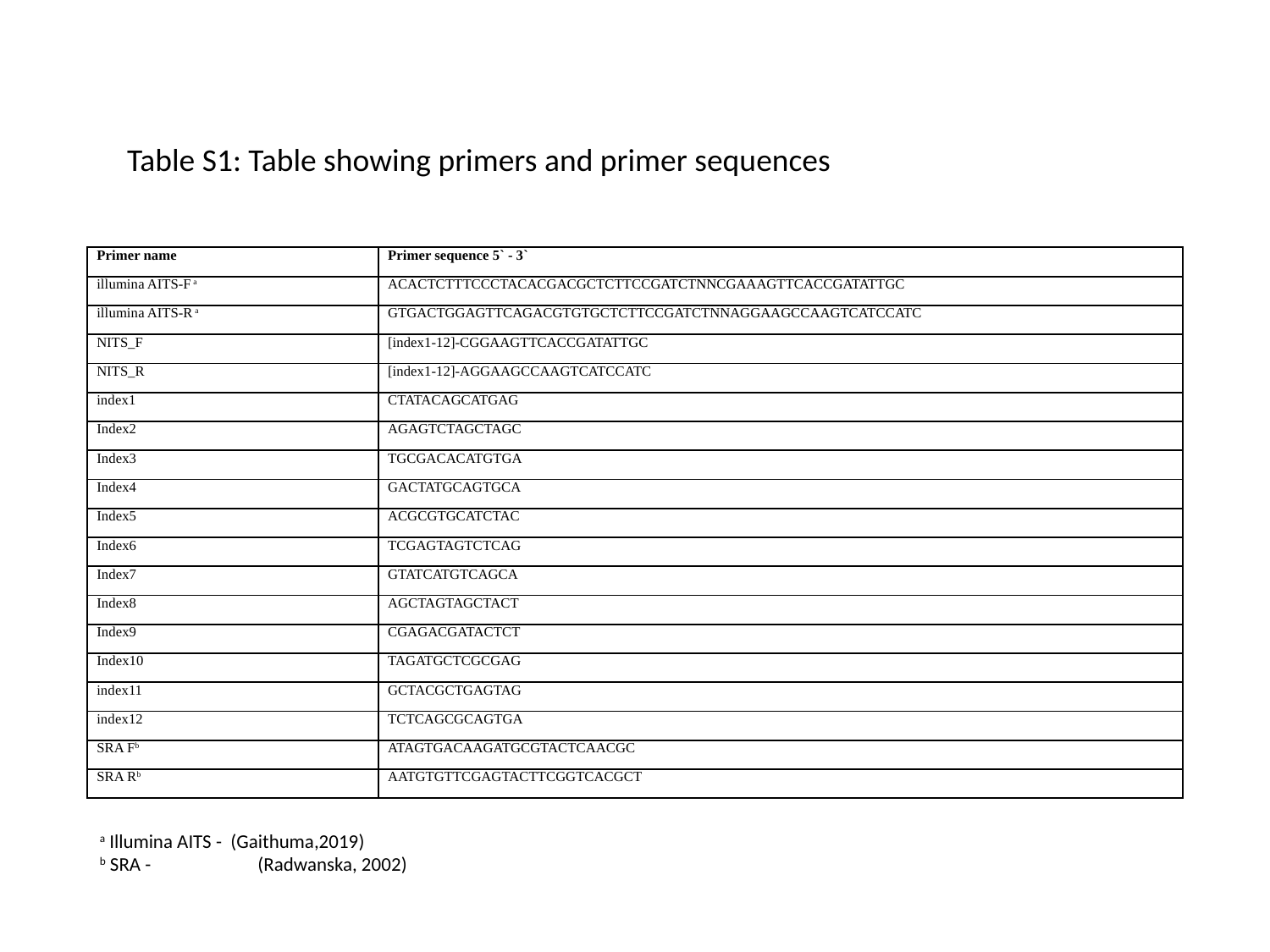

Table S1: Table showing primers and primer sequences
| Primer name | Primer sequence 5` - 3` |
| --- | --- |
| illumina AITS-F a | ACACTCTTTCCCTACACGACGCTCTTCCGATCTNNCGAAAGTTCACCGATATTGC |
| illumina AITS-R a | GTGACTGGAGTTCAGACGTGTGCTCTTCCGATCTNNAGGAAGCCAAGTCATCCATC |
| NITS\_F | [index1-12]-CGGAAGTTCACCGATATTGC |
| NITS\_R | [index1-12]-AGGAAGCCAAGTCATCCATC |
| index1 | CTATACAGCATGAG |
| Index2 | AGAGTCTAGCTAGC |
| Index3 | TGCGACACATGTGA |
| Index4 | GACTATGCAGTGCA |
| Index5 | ACGCGTGCATCTAC |
| Index6 | TCGAGTAGTCTCAG |
| Index7 | GTATCATGTCAGCA |
| Index8 | AGCTAGTAGCTACT |
| Index9 | CGAGACGATACTCT |
| Index10 | TAGATGCTCGCGAG |
| index11 | GCTACGCTGAGTAG |
| index12 | TCTCAGCGCAGTGA |
| SRA Fb | ATAGTGACAAGATGCGTACTCAACGC |
| SRA Rb | AATGTGTTCGAGTACTTCGGTCACGCT |
a Illumina AITS - (Gaithuma,2019)
b SRA -	 (Radwanska, 2002)

## Slide 2
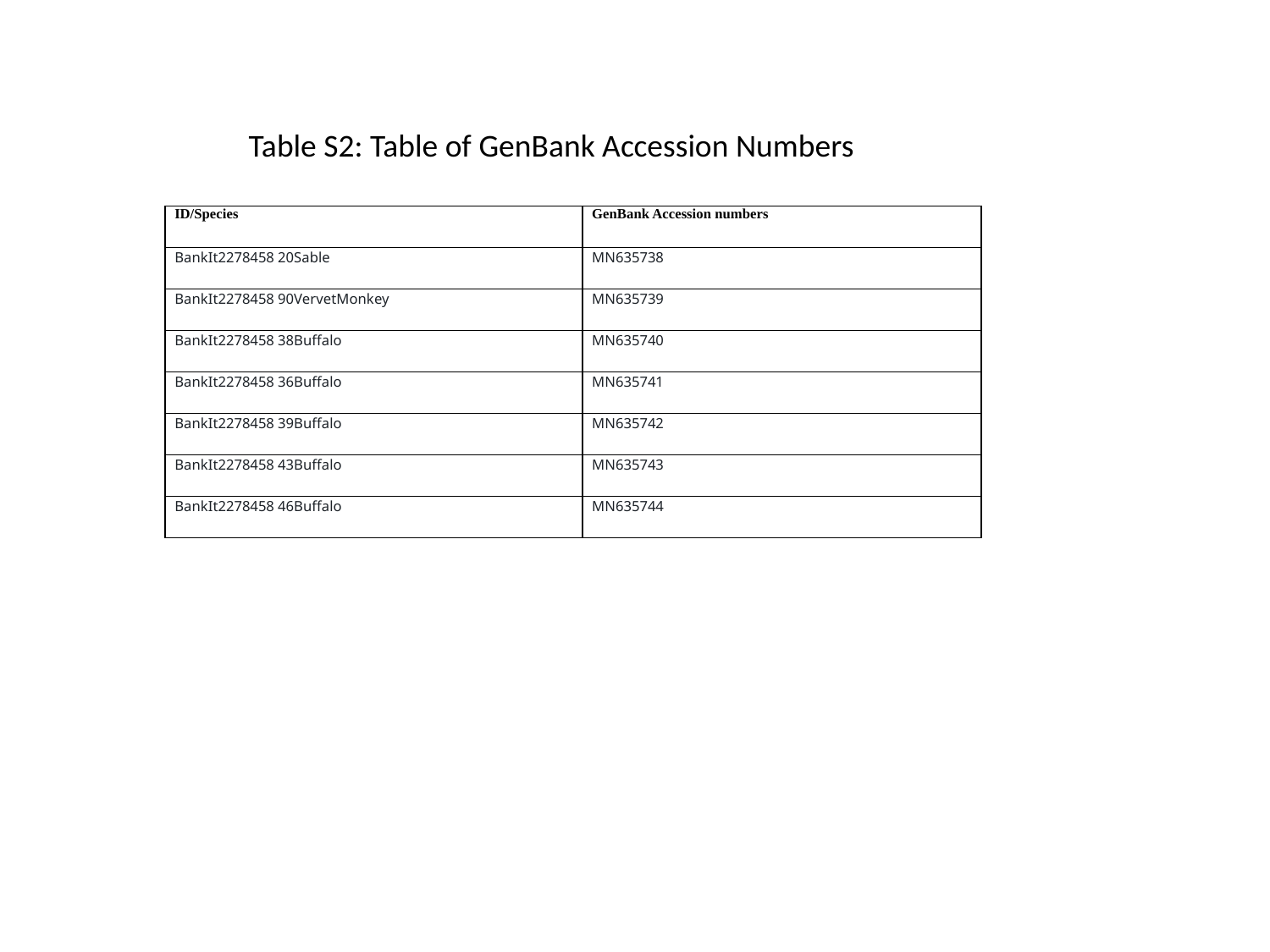

Table S2: Table of GenBank Accession Numbers
| ID/Species | GenBank Accession numbers |
| --- | --- |
| BankIt2278458 20Sable | MN635738 |
| BankIt2278458 90VervetMonkey | MN635739 |
| BankIt2278458 38Buffalo | MN635740 |
| BankIt2278458 36Buffalo | MN635741 |
| BankIt2278458 39Buffalo | MN635742 |
| BankIt2278458 43Buffalo | MN635743 |
| BankIt2278458 46Buffalo | MN635744 |

## Slide 3
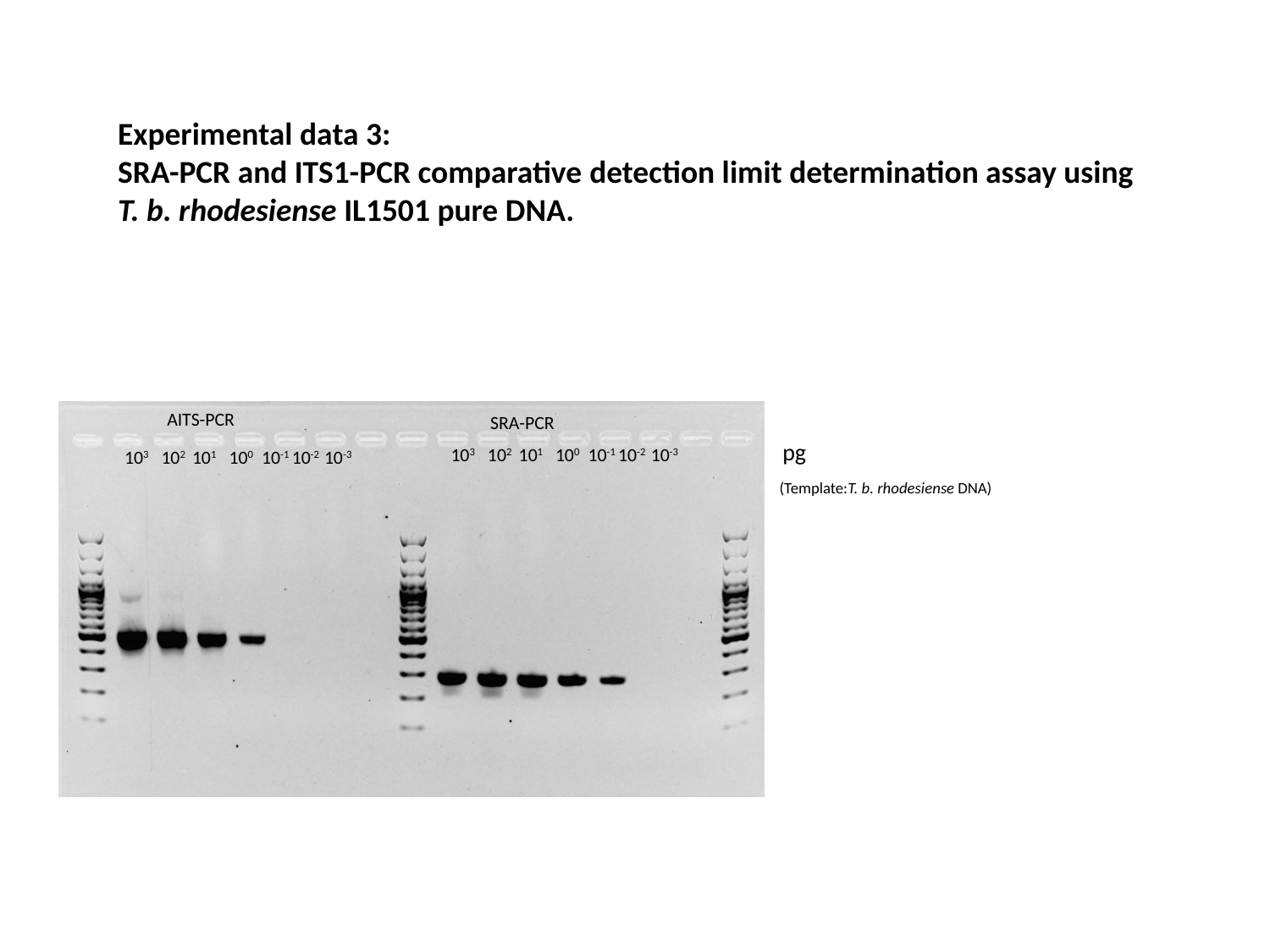

Experimental data 3:
SRA-PCR and ITS1-PCR comparative detection limit determination assay using T. b. rhodesiense IL1501 pure DNA.
AITS-PCR
SRA-PCR
pg
103 102 101 100 10-1 10-2 10-3
103 102 101 100 10-1 10-2 10-3
(Template:T. b. rhodesiense DNA)
